# The importance of habitat resistance for movement decisions in the common lizard, *Lacerta vivipara*

**DOI:** 10.1186/1472-6785-12-13

**Published:** 2012-07-24

**Authors:** Susanne RK Zajitschek, Felix Zajitschek, Jean Clobert

**Affiliations:** 1 Station d'Ecologie Expérimentale du CNRS a Moulis, USR 2936, 09200, Moulis, France; 2 Department of Evolutionary Biology, Evolutionary Biology Centre, Uppsala University, Norbyvägen 18D, 752 36, Uppsala, Sweden; 3 Department of Animal Ecology, Evolutionary Biology Centre, Uppsala University, Norbyvägen 18D, 752 36, Uppsala, Sweden

**Keywords:** Behaviour, Connectivity, Cover, Dispersal, Exploration, Humidity, Lacertidae, Light, Lizards, Personality, Phenotypic plasticity, Substrate, Temperature, Zootoca

## Abstract

**Background:**

Movement behaviour can be influenced by a multitude of biotic and abiotic factors. Here, we investigate the speed of movement in relation to environmental and individual phenotypic properties in subadult common lizards (*Lacerta vivipara*). We aim to disentangle the importance of substrate, cover, humidity, basking opportunity and individual phenotype on moving tendencies in 12 treatment combinations, at which each lizard was tested.

**Results:**

We find that movement behaviour depends on the starting conditions, the physical properties of the dispersal corridor, and on the individuals’ phenotype. Specifically, the presence of cover and substrate providing suitable traction in the corridor had positive effects on individual movement decisions. Additionally, we find high phenotypic variation in the propensity to move dependent on the presence of cover. Individual back patterns also strongly affected movement decisions in interaction with the physical properties of the dispersal corridor.

**Conclusions:**

Our results highlight the importance of understanding the habitat resistance for movement patterns, with humid habitats with covering vegetation providing the best conditions to initiate movement in the common lizard. In addition, population effects, differences in back pattern phenotype and individual plasticity were identified as key parameters influencing movement behaviour.

## Background

Dispersal and the tendency of animals to move away from their natal site at various stages in their life cycle has been extensively studied in a range of fields including population genetics, behavioural ecology and conservation ecology [1-4]. Traditionally, the dispersal process has been largely simplified: especially the emigration phase and the circumstances leadings to emigration (such as population density, population structure) as well as conditions upon arrival (immigration) have received a lot of attention [1,2,4,5]. Modelling of dispersal movements within metapopulations is a useful tool to predict movements and patch occupations, but often the connecting pathways, and thereby the movement behaviour itself, are largely ignored [1], but see [6-8]. Nonetheless, the act of dispersal or inter-patch movement in natural habitats presents dispersing individuals with highly variable habitat [2,7]. This landscape mosaic represents a range of habitats that may offer highly varied selection pressures for the dispersing individuals [9].

In order to allow meaningful and biologically relevant models to predict dispersal and long-term fluctuations in natural metapopulations, matrix-specific factors need to be identified in order to estimate connectivity appropriately. For this integration of metapopulation biology and landscape ecology, studies on habitat conductivity and permeability are necessary and useful tools. For example, a laboratory-based study by Stevens *et al.*[8] has investigated the permeabilities of different land covers for the movement of Natterjack toadlets (*Bufo calamita*), and provided evidence for differential resistance ratios, highlighting the connection between the animals’ ability to cross a given landscape and habitat-specific parameters. However, in general very little is known to date about the optimal properties of dispersal pathways and functional connectivity, and even less about the preferences of dispersers for a particular property. Notably, this is no easy task to study in nature. Particularly in non-mammal species, where the dispersal routes cannot be easily radio-tracked and retraced but often have to be estimated as shortest distance between capture and recapture points, the study of inter-patch movement remains challenging [10]. In addition, landscape properties and connectivity are expected to be highly species specific as there is a strong factor of scale [1,2,11] as well as size-dependent permeability of different habitat types [8]. Adding to the complexity is the fact that within-species variation is expected to be high, as it has been acknowledged recently that animal temperament may play an important role in ecological processes [12]*.* It seems to be particularly important in dispersal behaviour [2,4,5,12-15], and is expected to be consistent throughout the life cycle [4]. For example, bold individuals may be more inclined to be explorative, and thus more likely to move and disperse than shy individuals. This has been shown in firebugs (*Pyrrhocoris apterus*) [16], as well as in great tits (*Parus major*), where exploratory behaviour types were also shown to be heritable [17]. It has also been found that less social individuals are more likely to disperse, in mosquitofish (*Gambusia affinis*) [18] as well as in common lizards (*Lacerta vivipara* )[15]. In addition, it is possible that differences in personality or behaviour may also be manifest in morphological features, due to correlational selection on certain behaviours and phenotypic traits. This is the case for example in garter snakes (*Tamnophis ordinoides*) [19], where the linearity of back pattern is correlated with the modus of escape behaviour. There are also indications that in the common lizard certain life history strategies are correlated with dorsal pattern [20]; Miles et al, pers. com.]. The different dorsal patterns can be classified as either “linear” or “reticulated” morphs, which differ in the proportion of melanised areas. These are higher in linear morphs, which may have profound effects on optimal heating rates. In addition, linear individuals seem to follow a different ecological pathway than reticulated animals, which is evident in their slower growth and lower fecundity compared to reticulated individuals [20]. In addition, dorsal pattern is potentially linked to behaviour and dispersal, particularly in this species. Lepetz et al. [20] observed an increase of reticulated back patterns together with decreasing dispersal, across a time span of 11 years, coupled with rising temperatures in the field. These findings indicate that individuals bearing linear dorsal patterns are more likely to be dispersers, whereas reticulated animals may be representative of the resident behaviour type.

Here, we attempt to identify preferred dispersal pathway properties for the common lizard in a large-scale laboratory experiment, by examining movement tendencies across various physical channels, which we use as a proxy for dispersal propensity. It has been shown earlier that this lizard species displays different personalities, which can be connected to differing dispersal behaviour [4]. Natal dispersal usually occurs in this species at an early age (<10 days; [21]), but dispersal movements across distances that are typical for the study species (>30 meters; [22]) have been recorded in older juveniles as well [23-25]. Indeed, it has been shown that juvenile dispersal peaks again after natal dispersal (usually within the first year of life), depending on conditions [24]. Renewed bouts of dispersal have also been recorded in adult individuals in the study species following environmental perturbation and transplantation to novel locations [25]. Even though the fine-scaled effects of age on dispersal behaviour require further investigation, it can occur in the common lizards across all life stages. The rate of dispersal hereby strongly depends on individual motivation, which seems to be influenced by morphotypes [20] and personalities [4,15], which are stable throughout life [15]. In addition, habitat structures and their physical properties are likely to have similar effects on the movement behaviour of the animals, regardless of their exact age. This leads us to believe that our snapshot investigation of movement behaviour at the juvenile stage may be extendable to true dispersal movement in the here investigated species.

The animals occur commonly in bogs and humid grass- and heathlands across Europe and Asia. Their natural habitat contains diverse landscape components including ample cover and hiding space, as well as patches with direct sunlight for basking. To measure the connectivity between suitable habitats, and the resistance of the habitat matrix for this ground-dwelling species we have identified temperature, cover, substrate and humidity as most important variables (shown to influence dispersal, [22]), and hypothesize that different combinations of these components may provide diverging opportunities and advantages for movement and dispersal. We therefore measure the phenotypic plasticity of individuals to initiate movement in response to differing channels, displaying the fully factorial crossed variables, measuring both i) the time it takes the individuals to leave a starting environment under varying conditions and ii) how long the animal takes to cross corridors that display combinations of different physical properties. Specifically, we hypothesize that under undesirable conditions (no heating source) at the starting environment animals will tend to start moving faster than under favourable conditions (light and heat provided). We also predict that conditions that mimic natural habitat influence mobility positively, whereas unnatural (no substrate) or adverse conditions (dry substrate) may reduce speed of movement. In addition, the relative importance of the different factors and their interactions for movement behaviour are investigated.

## Results

**Figure 1:**
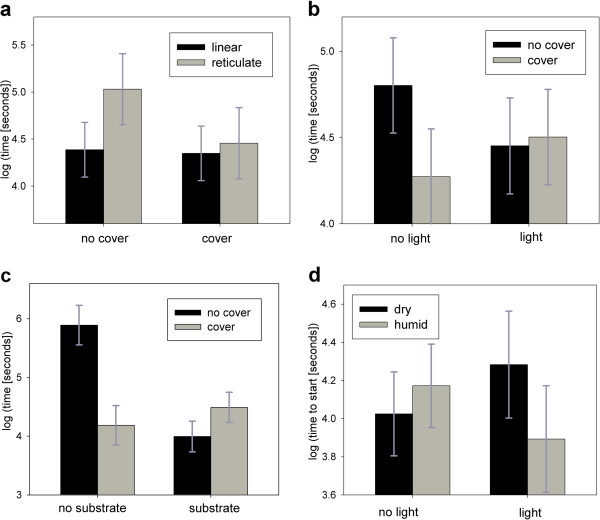
**Presentations of interactions for the time to finish (in seconds), *t *(1a: back pattern × cover; 1b: cover × light; 1c: cover × substrate) and time to start (in seconds), * ts *(1d: light × humidity). ** Bars represent means of fitted values on the log-transformed variable, using the final model (Table 1 for Figures 1a–c, Table 2 for 1**d **), error bars show their 95% confidence intervals.

We found that the time to cross the channel, *t*, was affected by both environmental treatment conditions and by the back pattern of an individual (Table 1), whereas the back pattern had no substantial explanatory power for *ts ,* the time it took an animal to start moving into the channel (Additional file 1: Table S2). The effect of back pattern morphotype on *t* depended substantially on cover (Table 1). Only in channels without cover did individuals with reticulate back pattern take substantially longer to cross the channel than individuals with linear back pattern (Figure 1a).

**Table 1:** **Final model for log(*t*), the time it took the animals to cross the channel and finish the trial**

**Model terms (reference level)**	**Effect value**	**CI**		**Df**
		**Lower**	**Upper**	
Light (no light)	−0.350	−0.661	−0.040	624
Substrate (no substrate)	−1.895	−2.269	−1.521	624
Cover (no cover)	−1.796	−2.266	−1.326	624
Humidity (dry)	−0.305	−0.587	−0.024	624
Population B (population A)	0.977	0.147	1.807	53
Population C (population A)	1.184	0.078	2.290	53
Population D (population A)	0.569	−0.306	1.444	53
Population C (population B)	0.207	−0.643	1.055	53
Population D (population B)	−0.408	−1.224	0.408	53
Population D (population C)	−0.615	−1.548	0.319	53
Back pattern (linear)	0.644	0.153	1.135	53
Light × Cover (no light × no cover)	0.580	0.140	1.021	624
Substrate × Cover (no substrate × no cover)	2.200	1.734	2.667	624
Cover × Back pattern (no cover × linear)	−0.536	−0.986	−0.085	624

Main and interaction model terms after model selection. “Reference level” gives factor value against which a model term was cross-checked. The population effects are represented as the results of multiple comparisons (details on method in Additional file).

CI: 95% confidence intervals; *Df:* degrees of freedom.

**Figure 2:**
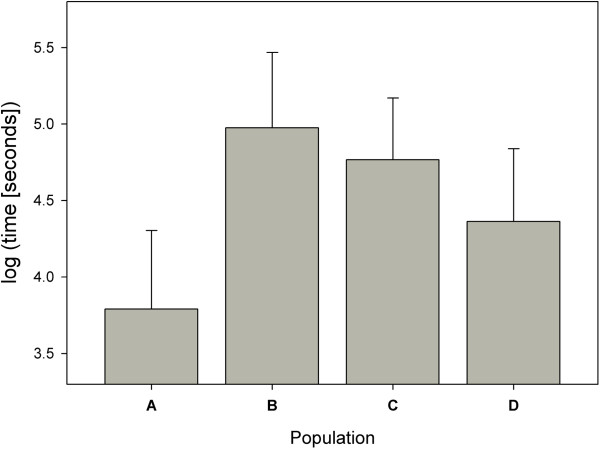
**Population differences in * t, *time to finish (in seconds), shown as means of fitted values on the log-transformed variable. ** Error bars represent 95% confidence intervals.

Dry substrate in the channel made lizards take about 42% longer to arrive at the receiving container, irrespective of other treatment variables (Table 1). The difference in *t* between lizards of different source populations was mainly caused by animals from one population crossing the channels faster than animals from two of the three other populations (population A, Table 1, Figure 2).

Animals were generally faster to cross the channel when no light and heat source was provided (Table 1).

Whether *t* was affected by the presence of a cover over the channel depended on the starting conditions (Table 1). Only when there was no light provided in the starting container, lizards took a smaller amount of time to arrive at the receiving container, when the channel was covered. With light, there was no effect of cover (Figure 1b). Similarly, we found no effect of substrate presence when the channel was covered, compared to a much longer time spent crossing an uncovered channel containing no substrate (Figure 1c).

*Ts* (the time it took a lizard to leave the starting compartment) was about 53% shorter when the channel was not covered (Table 2). In treatments where we provided light in the starting container, animals took longer to start inspecting the channel when the substrate in the channel was dry, compared to trials with wet substrate in the channel. Without light, this relationship was reversed (Table 2, Figure 1d).

**Table 2:** **Final model for log( *ts *), the time it took the animals to start entering the channels **

**Model terms (reference level)**	**Effect value**	**CI**		**Df**
		**Lower**	**Upper**	
Light (no light)	0.147	−0.095	0.389	628
Cover (no cover)	0.423	0.230	0.618	628
Humidity (dry)	0.259	−0.051	0.568	628
Light × Humidity (no light × dry)	−0.537	−0.950	−0.125	628

Main and interaction model terms after model selection. “Reference level” gives factor value against which a model term was cross-checked.

CI: 95% confidence intervals; Df: degrees of freedom.

We did not find any indication of individual flight responses on *ts*, as analyses in which we excluded *ts* < 10 seconds led to identical results as using the full dataset.

We found no effect of sex or personality trait (exploration or basking), as measured in the personality assays, on any time responses.

### Reaction norms

We found substantial variation in reaction norms between *t* of individuals in response to cover, as the model including random intercept and slope was superior to the model containing random intercept only (dAICc = 11.4). This was not found for *ts* (dAICc = 1.5). This means that (for *t*) cover considerably influenced phenotypic plasticity, leading to a much higher variety in individual movement patterns in trials where cover was present compared to trials with uncovered channels. Other variables had no substantial explanatory effect on reaction norm characteristics (dAICc < 2). The effect of cover on individual-level differences did not interact with other experimental variables. The variation in individual intercepts, which coincides in our case with individual responses to cover, was considerably higher than variation in individual-level differences in slopes (28% of total variance explained by random intercepts, compared to 11%).

## Discussion

We show that environmental characteristics that are likely to be important under natural conditions indeed affect movement decisions of juvenile common lizards in an experimental laboratory setting. Moreover, we find the back pattern type of the lizards to be a good predictor of movement behaviour under certain circumstances, and that population effects may play a role. In addition, we found high phenotypic plasticity in the movement behaviour with respect to the cover of the surroundings.

Specifically, we found a correlation of cover with back pattern type. Linear individuals seem to be bolder in overcoming the inhibiting effects of a habitat patch without cover, as seen in their faster movement through the channels compared to reticulate individuals. This matches the finding of higher immigration rates of linear individuals, compared to reticulate ones, in natural populations of this species [20]. This indicates that the different morphs in this species indeed may follow diverging life-history strategies, as there is already some evidence that individuals with linear dorsal patterns grow more slowly and live longer than reticulated morphs, and have a comparatively lower fecundity [20], Clobert, unpublished data]. Interestingly, the trend observed here of the more explorative and seemingly bolder linear individuals seems counterintuitive, as generally those individuals that are more willing to take risks are also expected to have matching life histories, such as rapid maturity, high fecundity and lower survival probabilities, which is more coherent with the life histories of reticulated lizards. However, it is possible that the measurements applied here do not necessarily reflect boldness and exploratory behaviour, if faster movement does not correspond with increased boldness. Indeed, it might quite be the opposite: linear individuals moved faster in order to avoid dangers such as predation in an unfamiliar environment, which was even exaggerated under unfavourable conditions (i.e. under conditions that were least similar to conditions of their natural habitats).

The fact that dispersal seems to be more common in animals with linear dorsal patterns [20] also points towards an explanation that is not necessarily linked to bold personality. The fast-lived life histories of the reticulated individuals might in fact be due to differential energy allocation: while linear individuals use their resources to disperse and settle into new habitats early in life, leading to reduced energy levels for growth and reproduction later on, reticulated lizards may make use of their natal sites, allocating all their energy into growth and higher or earlier fecundity with which it is associated.

Even though it is not entirely evident why movement decisions should be correlated with dorsal pattern morphotype, correlational selection for back pattern type and movement or dispersal behaviour constitutes a valid possibility. A correlation between back pattern and flight behaviour has been shown in garter snakes [19]. Striped garter snakes had higher survival when they took flight without showing a reversal of direction during the predator escape. For spotted snakes there was positive selection for more frequent change of directions during the escape. The adaptive significance of this example of correlational selection could be the perception of being slower when having a striped back pattern [26]. If the same were true for common lizards, reticulate individuals should be detected easier than linear individuals by visual predators, which could explain the more cautionary behaviour of reticulates. Fitting into this picture, reticulate common lizard females were found to stop more frequently during sprint speed performance trials on a race track, compared to linear individuals [D. Miles, *pers.com*.]. The latter study also found that reticulate females had higher endurance when tested on a treadmill. Taken together, the difference in behaviour between linear and reticulate common lizards indicate that there may be two distinct strategies of movement: linear individuals seem to move more between populations and are more likely or faster to make movement decisions, compared to animals bearing reticulate dorsal patterns.

Independent of dorsal pattern, the decision to move into the channel depended on whether the lizards were provided with a light and heat source in the starting container, in combination with humidity in the channel. As predicted, under circumstances where the animals that had been provided with a heat source, they took a longer time to enter the channels, given the channel was humid. Not astonishingly, a cold starting point, where no light and heat is provided, does not constitute a favourable environment and lizards can probably profit by searching for a more favourable patch. Indeed, thermoregulatory behaviour that makes use of available micro-habitat patches that differ in temperature and humidity has been shown in various lizard species [27,28]. In addition, individual differences in behaviour and specifically in temperament and personality are expected to affect the propensity to move (and disperse) as shown in a wide range of organisms [2,4,12,14,15]. However, here we did not find a strong link between individual personality and the speed to enter or move through a channel. This may indicate that the present design may not be useful to reflect true exploratory behaviour, and thus may be unsuitable to detect differences in personality, such as boldness. It is possible, that due to the acclimation to the laboratory, including maintenance and frequent handling during the trials, the lizards had already learned that the artificial environment in the laboratory is in fact predator-free, and represents relatively risk-limited surroundings. This may have led to a reduced expression of shyness or fear compared to conditions in the field, and potentially resulted in a narrower spectrum of reaction norms of individual differences in movement behaviour under all investigated circumstances, than may be found under natural conditions. However, in other studies on the same species it was found that exploratory and activity-related behavioural traits are correlated with the dispersal decision process [4,29]. There is therefore some possibility that these personality traits are either not involved in the transience phase of a dispersal process, or that the artificially created channels and the measurement of movement behaviour within are not related to dispersal decisions in the field. Importantly, we presently assume that the habitat properties that slow down movement as measured here will also affect the dispersal process negatively. However, it has been shown that this is not necessarily true, as boundary effects between differing substrate types and different attractiveness of the various substrates may be based on other criteria than movement facilitation [8,30]. This may lead to difficulties for the prediction of dispersal routes or tendency, and it is therefore possible that the here reported factors may not directly affect dispersal behaviour or the dispersal process in the common lizard.

Independent of the conditions in the starting container, lizards moved more quickly into the channel when the channel was not covered. The reason for slower entry into covered channels might be due to the novelty of the environment, paired with the greater inability to assess potential risks associated with moving into a dark refuge area. On the other hand, it is also possible that the lizards moved faster in open channels in their search for a refuge, to escape the open space in the starting container and uncovered channel, representing no protection from potential predators. However, the potential presence of a predator in a refuge or the cost of not being able to forage or bask while in the refuge may have influenced the individuals not to enter covered channels quickly. If the risk of staying outside the refuge is small, as in our design without chemical or visual cues of predators being present, the observed delay of moving into the covered channel may be predicted [31,32]. Nonetheless, we also found that individual variation in moving time in covered channels was much higher than in uncovered channels. This increased phenotypic plasticity may be favourable under certain ecological circumstances, and may allow animals that may not be likely to disperse otherwise, to colonise suitable habitat. On the other hand, it may also indicate that the animals may prefer to stay in refuges longer (than crossing open habitat) under certain circumstances, which are dependent on their current metabolic status. This is reflected in the interaction of light at starting conditions, and cover: when no light was provided in the starting container, animals took less time to cross covered channels than open channels. This might indicate that the animals that have not had the opportunity to thermoregulate were less inclined to stay inside the covered space, potentially in search of more favourable conditions (i.e. sunshine and opportunity to bask). Indeed, the fact that movement was faster when no basking opportunity had been provided in general may also point to the fact that the animals may try to evade unfavourable conditions. In cases where it took individuals a long time to cross the channels two factors may have played a major role: the speed of movement itself and the potential reluctance to continue moving (i.e. returning to the starting container after having initiated channel entry). However, we are unable to distinguish between slow rate of movement and returns to the home base from the data recorded in the current study. Even though we are unable to link the respective behaviours with for example dorsal pattern, both slow movement and frequent returns indicate a reluctance to move, and may be linked to less bold behaviour.

All investigated environmental variables had a substantial effect on the time to arrive at the receiving terrarium and finish a trial, once a lizard had inspected the channel. Humidity of the channel substrate had a direct effect, with lizards crossing the channel faster when the substrate was humid. This might indicate that the dry substrate may have posed a greater obstacle, maybe as a generally unfavoured environment, to any exploratory behaviour [see for example [22,33]. In general this finding is not surprising, as this lizard species occurs in humid, heterogeneous habitats [21]. Preferences for conditions that mimic their natural habitats may be imprinted in the animals, or may have evolved as local adaptations for the animals’ exploration and movement decisions. This may also be reflected by the observed population differences.

Interestingly, the presence of substrate in covered channels was not important. Only without cover, lizards took much longer time to arrive at the receiving container when no substrate was in the channel. This makes sense if the primary concern of the lizard is predation: if the terrain is covered and provides shelter from visual predators, moving is relatively safe. If there is no cover, predation risk is much higher and would be exaggerated even more by a surface without good traction (such as smooth plastic, as used here) which would potentially increase the time to escape.

## Conclusion

In conclusion, the above results imply that dry substrate, no cover (especially under unfavourable thermoregulatory conditions) or no substrate inhibit the propensity to explore and move to another habitat area. In other words, humid habitats with ample cover provide optimal conditions to initiate dispersal in this species. This study therefore highlights the importance of understanding the physical properties of the landscape mosaic for the study of dispersal [see also [2,7,8,30]. This knowledge will be essential for the long-term understanding and accurate prediction of metapopulation fluctuations in common lizards, and adds to the growing body of research investigating habitat resistance, conductivity, permeability and connectivity [for example [8,30]. In a world of increasing habitat destruction, decreasing connectivity and sinking population sizes, all factors influencing metapopulations need to be taken into account. As demonstrated here, specifically factors that influence the long neglected study of inter-patch dispersal need to be emphasised in our understanding of animal movement and dispersal.

## Methods

### Study species and experimental animals

Juvenile common lizards were captured between 20.06.2009 and 25.06.2009 from four different populations, from 2 km to 10 km apart, in the Cevennes (Mont Lozère, southern France, 44°27′ N, 3°44′ E). These distances between the populations are far enough apart to ensure that the sampled animals originated from separate populations, but provided almost identical climatic environments for the investigated populations at the same time.

We caught in total 63 animals (33 males and 30 females). Specifically, 16 animals (10 males, 6 females) were collected at population A, 16 animals (5 males, 11 females) at population B, 20 animals (14 males, 6 females) at population C, and 11 animals (4 males, 7 females) at population D. However, 4 animals had to be excluded from the analyses as they escaped during the trials and did not complete the full set of experiments.

We measured snout-vent length (SVL) to the nearest 0.1 mm and weighed animals to the nearest 0.001 g. Back pattern was scored as being either linear or reticulated [20]*.* We scored 10 linear / 6 reticulate animals in population A, 11 linear / 5 reticulate individuals in population B, 12 linear / 8 reticulate lizards in population C and 5 linear / 6 reticulate animals in population D.

Animals were maintained on an 8:16 light: dark cycle at a constant room temperature of 20°Celsius. We kept lizards in plastic containers (18 cm x 12 cm x 11 cm) and provided them with an egg carton (8 cm x 8 cm) for cover, and a combined light and heat source (CONCENTRA spot R63, 23 Watt, Osram, Munich, Germany). Lizards were fed every day, alternating between a small house cricket (*Acheta domesticus*) and two small meal worm larvae (*Tenebrio molitor*). We provided fresh water *ad libitum* in a small Petri dish, and the terraria were sprayed with water twice a day, to provide adequate humidity. For all experimentation, handling and maintenance the French Animal Ethics chart has been respected.

### Experimental design

#### Personality assays

First, we observed what kind of behaviour experimental animals showed when put individually in a terrarium (35 cm x 18 cm x 22 cm) that contained 3 cm of earth as substrate, together with two pieces of egg cartons (8 cm x 8 cm x 5 cm), placed at the opposite longer sides, as hiding shelters. In the first set of trials, we provided no heat or spot light source and the ambient temperature was held at 20°Celsius. After a 2-minute acclimation time we scored every 10 seconds whether an individual was immobile (without hiding), hiding under an egg carton, or exploring, for 10 minutes. For each trial, new substrate and fresh egg cartons were provided. This first experiment gave us individual estimates of the tendency to explore a new environment without a heat or light source which we will refer to as ‘exploration tendency’ from here on. In a second set of trials, which were started 1 h after the end of the first trial, we added a light and heat source (25 W Osram Concentra lamp, 20 cm above the surface), and used the same protocol as in the first set of trials, except that ‘basking’ was added to the list of observable behaviours. The time an animal spent basking was used as a measure of its boldness [see [34]. Variables exploration tendency and boldness are referred to as traits describing part of the personality of the experimental animals.

#### Physical properties influencing movement preferences

In the following part of this study, we employed a cross-over design in which each animal was tested in 12 trials (corresponding to 12 treatments, one treatment per day) in a randomly assigned order, resulting in a total sample size of 708 observations. For this, we built experimental units consisting of two terraria (18 cm x 12 cm x 11 cm) that were connected with a channel (80 cm x 5 cm, with 10 cm high sidewalls) made out of clear plastic sheet that was open at the top. Home ranges of the species average 10 m, and dispersal distances are >30 m [15,22]. However, we believe that the effects of the habitat resistance should be to some extent independent of scale and therefore may predict movement behaviour that may be related to the dispersal process, even in 80 cm long pathways. The opening through which a lizard was able to enter and exit each terrarium, and move into and out of the channel, was 2 cm x 2 cm large and at a same level position as the surface of the terrarium and the channel. This allowed for a seamless movement between terraria and channel, unimpeded by any height inequalities of the surface. We manipulated light/heat, substrate, cover and humidity conditions as follows. For light/heat conditions, there either was a lamp (‘with light’; 25 W Osram Concentra lamp, 20 cm above the surface) or no lamp (‘no light’) provided in the starting terrarium. In the other three treatments we manipulated conditions in the dispersing channel only. Channels either contained substrate (‘with substrate’) or no substrate (‘no substrate’), the substrate was either wetted before the trial (‘humid’) or left dry (‘dry’), and channels were either covered (‘with cover’) or left open (‘no cover’). For the cover treatment, we put a grey opaque plastic pipe (radius r = 5 cm), cut in half lengthwise, in the channel so that the whole channel length was covered and completely shaded. We crossed treatments in a factorial way, except substrate and humidity: when there was no substrate in the channel, we always left the channel dry, as it was not possible to get a sufficiently uniform distribution of the water on the smooth plastic surface. This resulted in 12 treatment groups in total (instead of 16 for the full factorial crossing). Before the beginning of each trial, we slightly wetted the substrate in the starting terrarium by spraying a specific amount of water on it. At the start of each trial, an individual lizard was put in the starting terrarium that always contained 1 cm of earth as substrate, without any other structures. We measured the time it took the lizard to find the opening to the channel and move at least its head completely into the channel (starting time*, ts*). Time *t* (time to finish) was the time between when the individual first put its head into the channel (i.e. ts) and when it arrived into the receiving terrarium. Hence time to finish included cases where a lizard would move right through the channel, and cases in which it did not move all the way through but went back to the starting terrarium or stayed somewhere in the channel, until it arrived at the receiving terrarium. All experimental animals crossed the channels in all trials, with the upper 5% having a mean of 97 minutes for *t*, and 22 minutes for *ts*.

### Statistical analysis

#### Morphological measurements

Weight and SVL were highly correlated; therefore we used the first principal component that explained 91.9% of variation in these two variables, as a composite size estimate in further analyses (pc1size, see Additional file 1).

#### Channel preferences

We investigated the effects of treatment (light, cover, substrate, humidity), the effects of sex, back pattern and personality of an individual, and its source population. We also included two-way interaction effects between treatments, and between treatment and the variables sex, back pattern and population, using linear mixed models implemented in the nlme package in the software R [35]. For a detailed formulation of the global models, and for model selection procedure using AICc, please see Additional file 1. To calculate the percentage change of the time response on the original untransformed scale, we used the formula 100 x (exp(*β*) – 1), *β* being the model coefficient.

To examine phenotypic reaction norms in movement responses, i.e. how plastic the responses were to experimental treatments among individuals, we compared models where the individual slopes (i.e behaviour) were either constant (i.e. random intercept only) with those including individual effects conditioned on any of the treatment variables (i.e. including random slope). For this, we used mixed models in the lme4 package in R [36]. For models with substantial variation in individual-based slopes, we also examined whether the observed random effect on slope depended on other variables, by analysing interaction effects.

## Competing interests

The authors declare that they have no competing interests.

## Authors’ contributions

SZ and FZ designed and performed the experiments. JC originally formulated the idea. FZ analysed the data. SZ wrote the manuscript; FZ and JC provided editorial advice. All authors read and approved the final manuscript.

## Supplementary Material

Additional file 1 **Supporting information (Zajitschek et al. 2012).** 1. Formulation of the global model and explanation of the model selection procedure. 2. Table S1: Model selection for *t.* 3. Table S2: Model selection for *ts.*[37,38]. Click here for file

## References

[B1] Wiens JA Clobert J, Danchin E, Dhondt AA, Nichols JD The landscape context of dispersal Dispersal 2001 United Kingdom: Oxford University Press 96 109

[B2] Bowler DE Benton TG Causes and consequences of animal dispersal strategies: relating individual behaviour to spatial dynamics Biol Rev 2005 80 2 205 225 10.1017/S1464793104006645 15921049

[B3] Kokko H López-Sepulcre A From individual dispersal to species ranges: perspectives for a changing world Science (New York, NY) 2006 313 789 791 10.1126/science.1128566 16902127

[B4] Cote J Clobert J Brodin T Fogarty S Sih A Personality-dependent dispersal: characterization, ontogeny and consequences for spatially structured populations Philos Trans R Soc B Biol Sci 2010 365 1560 4065 4076 10.1098/rstb.2010.0176 PMC2992741 21078658

[B5] Clobert J Le Galliard JF Cote J Meylan S Massot M Informed dispersal, heterogeneity in animal dispersal syndromes and the dynamics of spatially structured populations Ecol Lett 2009 12 3 197 209 10.1111/j.1461-0248.2008.01267.x 19170731

[B6] Revilla E Wiegand T Individual movement behavior, matrix heterogeneity, and the dynamics of spatially structured populations Proc Natl Acad Sci U S A 2008 105 19120 19125 10.1073/pnas.0801725105 19060193 PMC2614725

[B7] Revilla E Wiegand T Palomares F Ferreras P Delibes M Effects of matrix heterogeneity on animal dispersal: From individual behavior to metapopulation-level parameters Am Nat 2004 164 5 E130 E153 10.1086/424767 15540147

[B8] Stevens VM Polus E Wesselingh RA Schtickzelle N Baguette M Quantifying functional connectivity: experimental evidence for patch-specific resistance in the Natterjack toad *(Bufo calamita)* Landsc Ecol 2004 19 829 842 10.1007/s10980-004-0166-6

[B9] Wiens JA Gardner RH, Kemp WM, Kennedy VS, Petersen JE Understanding the problem of scale in experimental ecology Scaling Relations in Experimental Ecology 2001 61 88

[B10] Morales JM Moorcroft PR Matthiopoulos J Frair JL Kie JG Powell RA Merrill EH Haydon DT Building the bridge between animal movement and population dynamics Philos Trans R Soc B Biol Sci 2010 365 1550 2289 2301 10.1098/rstb.2010.0082 PMC2894961 20566505

[B11] Baguette M VanDyck H Landscape connectivity and animal behavior: functional grain as a key determinant for dispersal Landsc Ecol 2007 22 1117 1129 10.1007/s10980-007-9108-4

[B12] Réale D Reader SM Sol D McDougall PT Dingemanse NJ Integrating animal temperament within ecology and evolution Biol Rev Camb Philos Soc 2007 82 291 318 10.1111/j.1469-185X.2007.00010.x 17437562

[B13] Clobert J Ims R Rousset F Hanski I, Gaggiotti OE Causes, mechanisms and consequences of dispersal Ecology, Genetics and Evolution of Metapopulations 2004 San Diego, USA: Academic Press 307 335

[B14] Vandyck H Baguette M Dispersal behaviour in fragmented landscapes: Routine or special movements? Basic Appl Ecol 2005 6 535 545 10.1016/j.baae.2005.03.005

[B15] Cote J Clobert J Social personalities influence natal dispersal in a lizard Proc Biol Sci/R Soc 2007 274 383 390 10.1098/rspb.2006.3734 PMC1702372 17164202

[B16] Gyuris E Feró O Tartally A Barta Z Individual behaviour in firebugs *(Pyrrhocoris apterus)* Proc Biol Sci/R Soc 2010 628 633 10.1098/rspb.2010.1326 PMC3025677 20826482

[B17] Dingemanse NJ Both C van Noordwijk AJ Rutten AL Drent PJ Natal dispersal and personalities in great tits *(Parus major)* Proc Biol Sci/R Soc 2003 270 741 747 10.1098/rspb.2002.2300 PMC1691302 12713749

[B18] Cote J Fogarty S Weinersmith K Brodin T Sih A Personality traits and dispersal tendency in the invasive mosquitofish *(Gambusia affinis)* Proc Biol Sci/R Soc 2010 277 1571 1579 10.1098/rspb.2009.2128 PMC2871838 20071380

[B19] Brodie ED Correlational selection for color pattern and antipredator behaviour in the garter snake *Thamnophis ordinoides* Evolution 1992 46 1284 1298 10.2307/2409937 28568995

[B20] Lepetz V Massot M Chaine AS Clobert J Climate warming and the evolution of morphotypes in a reptile Glob Chang Biol 2009 15 454 466 10.1111/j.1365-2486.2008.01761.x

[B21] Clobert J Massot M Lecomte J Sorci G Defraipont M Barbault R Determinants of dispersal behavior - the common lizard as a case study Lizard Ecology: Historical and Experimental Perspectives 183 206

[B22] Massot M Clobert J Lorenzon P Rossi JM Condition-dependent dispersal and ontogeny of the dispersal behaviour: an experimental approach J Anim Ecol 2002 71 2 253 261 10.1046/j.1365-2656.2002.00592.x

[B23] Cote J Clobert J Risky dispersal: avoiding kin competition despite uncertainty Ecology 2010 91 5 1485 1493 10.1890/09-0387.1 20503880

[B24] Le Galliard JF Ferriere R Clobert J Mother-offspring interactions affect natal dispersal in a lizard Proc R Soc London, Ser B 2003 270 1520 1163 1169 10.1098/rspb.2003.2360 PMC1691359 12816655

[B25] Massot M Clobert J Pilorge T Lecomte J Barbault R Density dependence in the common lizard - demographic consequences of a density manipulation Ecology 1992 73 5 1742 1756 10.2307/1940026

[B26] Jackson JF Iii WI Campbell HW The dorsal pigmentation pattern of snakes as an antipredator strategy: a multivariate approach Am Nat 1976 110 1029 1053 10.1086/283125

[B27] Huey RB Pianka ER Seasonal variation in thermoregulatory behavior and body temperature of diurnal Kalahari lizards Ecology 1977 58 1066 1075 10.2307/1936926

[B28] Castilla AM Bauwens D Thermal biology, microhabitat selection, and conservation of the insular lizard Podarcis hispanica atrata Oecologia 1991 85 366 374 10.1007/BF00320612 28312041

[B29] De Fraipont M Clobert J John H Alder S Increased pre-natal maternal corticosterone promotes philopatry of offspring in common lizards *Lacerta vivipara* J Anim Ecol 2000 69 3 404 413 10.1046/j.1365-2656.2000.00405.x

[B30] Stevens VM Leboulenge E Wesselingh RA Baguette M Quantifying functional connectivity: experimental assessment of boundary permeability for the natterjack toad *(Bufo calamita)* Oecologia 2006 150 1 161 171 10.1007/s00442-006-0500-6 16896772

[B31] Amo L López P Martín J Refuge use: A conflict between avoiding predation and losing mass in lizards Physiol Behav 2007 90 334 343 10.1016/j.physbeh.2006.09.035 17109901

[B32] Cooper WE Wilson DS Beyond optimal escape theory: microhabitats as well as predation risk affect escape and refuge use by the phrynosomatid lizard Sceloporus virgatus Behaviour 2007 144 1235 1254 10.1163/156853907781890940

[B33] Lorenzon P Clobert J Massot M The contribution of phenotypic plasticity to adaptation in Lacerta *vivipara* Evolution 2001 55 392 11308095 10.1111/j.0014-3820.2001.tb01302.x

[B34] Cote J Dreiss A Clobert J Social personality trait and fitness Proc R Soc B Biol Sci 2008 275 1653 2851 2858 10.1098/rspb.2008.0783 PMC2605833 18755678

[B35] Pinheiro J Bates D DebRoy S Sarkar D R.C.Team nlme: Linear and nonlinear mixed effects models 2009 1 96 R package version 3

[B36] Bates D Maechler M lme4: Linear mixed-effects models using S4 classes 2009 R package version 0.999375-32

[B37] Diaz-Uriarte R Incorrect analysis of crossover trials in animal behaviour research Anim Behav 2002 63 815 822 10.1006/anbe.2001.1950

[B38] Hothorn T Bretz F Westfall P Simultaneous inference in general parametric models Biom J 2008 50 3 346 363 10.1002/bimj.200810425 18481363

