# Supplementary material for: The importance of habitat resistance for movement decisions in the common lizard, Lacerta vivipara
Source: BMC Ecol. 2012 Jul 24;12:13. doi: 10.1186/1472-6785-12-13 (PMC3488003; doi:10.1186/1472-6785-12-13)
Supplement: Additional file 1 — Supporting information (Zajitschek et al. 2012). 1. Formulation of the global model and explanation of the model selection procedure. 2. Table S1: Model selection for t. 3. Table S2: Model selection for ts.[37,38]. [file 1472-6785-12-13-S1.doc]

**1. Formulation of the global model and explanation of the model selection procedure.**

Global Model used to model *t* and *ts*:

log(*y*) = *µ* + light + substrate + cover + humidity +

light × substrate + light × cover + light × humidity + substrate × cover + cover × humidity +

sex + backpattern + population + personality trait +

+ population × light + population × substrate + pop × cover + pop × humidity +

+ sex × light + sex × substrate + sex × cover + sex × humidity +

+ backpattern × light + backpattern × substrate + backpattern × cover + backpattern × humidity +

pc1size + period + firstperiod + co_light + co_substrate + co_cover+ co_humidity + id + ε

where *y* was either *t* or *ts*. In order to meet the condition of a normal distribution of the residuals of the response variables *t* and *ts*, all two separate model selection procedures were conducted on models with the logarithm of these variables.

The intercept is given as *µ.* Treatment variables are fixed effects, including their two-way interactions. Sex, backpattern, population and personality trait were also fixed effects, where population gives the population in which the animal was captured and personality trait was either exploration tendency or boldness (investigated in separate models). Variable pc1size was kept in all analyses, as were variables period, firstperiod, co_light, co_substrate , co_cover, and co_humidity.

The latter six variables were included to account for habituation effects and modelled the calendar date, and therefore the sequence of trials for each individual (period), the trial that was conducted first (firstperiod), and the carry over (co) effects of the preceding trial (coded as 1 if the same treatment was analysed in the previous trial, or 0 otherwise) [37].

Individual identity, id, was included as a random effect.

To select the model that was supported most, we used Akaike’s Information Criterion adjusted for smaller sample sizes (AICc), with smaller AICc values indicating a better model fit (using AIC instead of AICc gave qualitatively the same results). To compare models, differences in AICc (dAICc) between models were calculated, and a difference of > 2 was taken to signify a substantial difference in model fit between two models (Burnham & Anderson 2002). Values of dAICc were calculated and are presented as (higher AICc) – (lower AICc ) in the main text.

Results of model selection were qualitatively not different when we used boldness instead of exploration tendency, therefore we only present dAICc values for models including boldness as a personality trait from hereon.

To select and retain important fixed effect in models, we first compared models without interaction effects of either population, sex, or back pattern with treatment variables, against the global model (e.g. without interactions including sex: sex × light + sex × substrate + sex × cover + sex × humidity). If the reduced model provided a better fit, we proceeded to compare models without the interaction terms separately against the global model. When the exclusion of a specific set of interaction terms gave a worse fit than the global model, we retained these interaction terms and proceeded by excluding single interaction terms (out of the retained set) in further models. If exclusion of a set of interaction terms did not result in a decrease in model fit, we excluded this set of interaction terms and used the reduced model as new baseline model for further comparisons.

After investigating interaction terms, we analysed main effects in the same way. If a main effect was part of an important interaction term, it was not evaluated on its own.

The chosen final models contain the least number of variables among statistically equal models (models listed above the dotted line, tables S1, S2). When the difference in AICc between two models is less than 2, there is no statistical basis for choosing among them.

The final model for *t* contained the independent categorical variable ‘population’. We used all-pairwise comparisons of Tukey in the R package multcomp [38] to estimate effect values and confidence intervals between populations (Table 1).

Table S1. Model selection for *t*.

| **#** | **L** | **S** | **C** | **H** | **Pop** | **Sex** | **Bp** | Pt | L*S | **L*C** | **L*H** | **S*C** | **C*H** | **L*Pop** | **S*Pop** | **C*Pop** | **H*Pop** | **L*Sex** | S*Sex | **C*Sex** | **H*Sex** | **L*Bp** | **S*Bp** | **C*Bp** | **H*Bp** | **AICc** | **ΔAICc** |
| --- | --- | --- | --- | --- | --- | --- | --- | --- | --- | --- | --- | --- | --- | --- | --- | --- | --- | --- | --- | --- | --- | --- | --- | --- | --- | --- | --- |
| **1** | x | x | x | x | x |  | x |  |  | x |  | x |  |  |  |  |  |  |  |  |  |  | x | x |  | 2677.20 | 0 |
| **2** | x | x | x | x | x | x | x | x | x | x |  | x | x |  |  |  |  |  |  |  |  |  | x | x |  | 2677.80 | 0.59 |
| **3^†^** | x | x | x | x | x |  | x |  |  | x |  | x |  |  |  |  |  |  |  |  |  |  |  | x |  | 2678.22 | 1.01 |
| **4** | x | x | x | x | x | x | x |  |  | x |  | x |  |  |  |  |  |  |  |  |  |  | x | x |  | 2678.79 | 1.59 |
| **5** | x | x | x | x | x |  | x | x |  | x |  | x |  |  |  |  |  |  |  |  |  |  | x | x |  | 2679.34 | 2.14 |
| **6** | x | x | x |  |  |  | x | x |  | x |  | x |  |  |  |  |  |  |  |  |  |  | x | x |  | 2679.60 | 2.39 |
| **7** | x | x | x | x | x | x | x | x | x | x | x | x | x |  |  |  |  |  |  |  |  |  | x | x |  | 2679.78 | 2.57 |
| **8** | x | x | x | x | x | x | x | x | x | x | x | x |  |  |  |  |  |  |  |  |  |  | x | x |  | 2680.11 | 2.9 |
| **9** | x | x | x | x | x | x | x | x |  | x | x | x | x |  |  |  |  |  |  |  |  |  | x | x |  | 2680.50 | 3.29 |
| **10** | x | x | x | x | x | x | x | x |  | x |  | x |  |  |  |  |  |  |  |  |  |  | x | x |  | 2680.96 | 3.76 |
| **11** | x | x | x | x | x | x | x | x | x | x | x | x | x |  |  |  |  |  |  |  |  |  | x | x | x | 2681.44 | 4.24 |
| **12** | x | x | x | x | x | x | x | x | x | x | x | x | x |  |  |  |  |  |  |  |  | x | x | x |  | 2681.66 | 4.45 |
| **13** | x | x | x | x | x | x | x | x | x | x | x | x | x |  |  |  |  |  |  |  |  | x | x | x | x | 2683.32 | 6.12 |
| **14** | x | x | x | x | x | x | x | x | x |  | x | x | x |  |  |  |  |  |  |  |  |  | x | x |  | 2684.06 | 6.86 |
| **15** | x | x | x | x |  | x | x | x |  | x |  | x |  |  |  |  |  |  |  |  |  |  | x | x |  | 2684.44 | 7.23 |
| **16** | x | x | x | x | x | x | x | x | x | x | x | x | x |  |  |  |  |  |  |  |  | x |  | x | x | 2684.97 | 7.77 |
| **17** | x | x | x | x | x | x | x | x | x | x | x | x | x |  |  |  |  |  |  |  |  | x | x |  | x | 2686.36 | 9.15 |
| **18** | x | x | x | x | x | x | x | x | x | x | x | x | x |  |  |  |  | x | x | x | x | x | x | x | x | 2691.34 | 14.14 |
| **19** | x | x | x | x | x | x | x | x | x | x | x | x | x | x | x | x | x |  |  |  |  | x | x | x | x | 2700.97 | 23.77 |
| **20** | x | x | x | x | x | x | x | x | x | x | x | x | x | x | x | x | x | x | x | x | x | x | x | x | x | 2708.97 | 31.77 |
| **21** | x | x | x | x | x | x | x | x | x | x | x | x | x | x | x | x | x | x | x | x | x |  |  |  |  | 2711.19 | 33.98 |
| **22** | x | x | x | x | x | x | x | x | x | x | x |  | x |  |  |  |  |  |  |  |  |  | x | x |  | 2729.32 | 52.12 |
| **23** |  |  |  |  |  |  |  |  |  |  |  |  |  |  |  |  |  |  |  |  |  |  |  |  |  | 2806.06 | 128.85 |

Abbreviations: #: model number; L: light; S: substrate; C: cover; H: humidity; Pop: population; Bp: back pattern; Pt: personality trait; interactions are depicted by the symbol *, final model by **^†^**

Table S2. Model selection for *ts*.

| **#** | **L** | **S** | **C** | **H** | **Pop** | **Sex** | **Bp** | Pt | L*S | **L*C** | **L*H** | **S*C** | **C*H** | **L*Pop** | **S*Pop** | **C*Pop** | **H*Pop** | **L*Sex** | S*Sex | **C*Sex** | **H*Sex** | **L*Bp** | **S*Bp** | **C*Bp** | **H*Bp** | **AICc** | **ΔAICc** |
| --- | --- | --- | --- | --- | --- | --- | --- | --- | --- | --- | --- | --- | --- | --- | --- | --- | --- | --- | --- | --- | --- | --- | --- | --- | --- | --- | --- |
| **1^†^** | x |  | x | x |  |  |  |  |  |  | x |  |  |  |  |  |  |  |  |  |  |  |  |  |  | 2480.14 | 0 |
| **2** | x | x | x | x |  |  |  |  |  |  | x |  |  |  |  |  |  |  |  |  |  |  |  |  |  | 2482.06 | 1.92 |
| **3** | x | x | x | x |  | x | x | x |  |  | x |  |  |  |  |  |  |  |  |  |  |  |  |  |  | 2485.78 | 5.64 |
| **4** | x | x | x | x | x | x |  | x |  |  | x |  |  |  |  |  |  |  |  |  |  |  |  |  |  | 2487.53 | 7.40 |
| **5** | x | x | x | x | x |  | x | x |  |  | x |  |  |  |  |  |  |  |  |  |  |  |  |  |  | 2487.91 | 7.78 |
| **6** | x | x | x | x | x | x | x |  |  |  | x |  |  |  |  |  |  |  |  |  |  |  |  |  |  | 2488.20 | 8.06 |
| **7** | x | x | x | x | x | x | x | x |  |  | x |  |  |  |  |  |  |  |  |  |  |  |  |  |  | 2489.11 | 8.97 |
| **8** | x | x | x | x | x | x | x | x | x | x | x | x |  |  |  |  |  |  |  |  |  |  |  |  |  | 2493.22 | 13.09 |
| **9** | x | x | x | x | x | x | x | x |  | x | x | x | x |  |  |  |  |  |  |  |  |  |  |  |  | 2493.24 | 13.10 |
| **10** | x | x | x | x | x | x | x | x | x | x | x |  | x |  |  |  |  |  |  |  |  |  |  |  |  | 2493.57 | 13.43 |
| **11** | x | x | x | x | x | x | x | x | x |  | x | x | x |  |  |  |  |  |  |  |  |  |  |  |  | 2495.00 | 14.86 |
| **12** | x | x | x | x | x | x | x | x | x | x | x | x | x |  |  |  |  |  |  |  |  |  |  |  |  | 2495.41 | 15.27 |
| **13** |  |  |  |  |  |  |  |  |  |  |  |  |  |  |  |  |  |  |  |  |  |  |  |  |  | 2496.12 | 15.98 |
| **14** | x | x | x | x | x | x | x | x | x | x |  | x | x |  |  |  |  |  |  |  |  |  |  |  |  | 2497.64 | 17.50 |
| **15** | x | x |  | x |  |  |  |  |  |  | x |  |  |  |  |  |  |  |  |  |  |  |  |  |  | 2497.78 | 17.64 |
| **16** | x | x | x | x | x | x | x | x | x | x | x | x | x |  |  |  |  | x | x | x | x | x | x | x | x | 2508.66 | 28.52 |
| **17** | x | x | x | x | x | x | x | x | x | x | x | x | x | x | x | x | x |  |  |  |  | x | x | x | x | 2520.64 | 40.50 |
| **18** | x | x | x | x | x | x | x | x | x | x | x | x | x | x | x | x | x | x | x | x | x |  |  |  |  | 2522.49 | 42.36 |
| **19** | x | x | x | x | x | x | x | x | x | x | x | x | x | x | x | x | x | x | x | x | x | x | x | x | x | 2529.07 | 48.93 |

Abbreviations: #: model number; L: light; S: substrate; C: cover; H: humidity; Pop: population; Bp: back pattern; Pt: personality trait; interactions are depicted by the symbol *, final model by **^†^**
